# Prospective associations of prenatal stress with child behavior: Moderation by the early childhood caregiving environment

**DOI:** 10.1017/S0954579424000920

**Published:** 2024-05-13

**Authors:** Gabrielle R. Rinne, Mallory Podosin, Nicole E. Mahrer, Madeleine U. Shalowitz, Sharon Landesman Ramey, Christine Dunkel Schetter

**Affiliations:** 1 University of California, Los Angeles, CA, USA; 2 University of La Verne, La Verne, CA, USA; 3 Rush University Medical Center, Chicago, IL, USA; 4 Virginia Polytechnic Institute and State University, Blacksburg, VA, USA

**Keywords:** Home environment, inhibitory control, mental health, parenting, prenatal stress

## Abstract

Fetal exposure to prenatal stress can increase risk for psychopathology but postnatal caregiving may offset risk. This study tests whether maternal sensitivity and the home environment during early childhood modify associations of prenatal stress with offspring behavior in a sample of 127 mother–child pairs (*n* = 127). Mothers reported on perceived stress during pregnancy. Maternal sensitivity was rated by coders during a parent–child free play task when children were 4 years old. One year later, mothers reported on the home environment, child internalizing and externalizing behaviors, and children completed an assessment of inhibitory control. As hypothesized, the early childhood caregiving environment modified associations of prenatal stress with child behavior. Specifically, prenatal stress was associated with more internalizing behaviors at lower levels of maternal sensitivity and in home environments that were lower in emotional support and cognitive stimulation, but not at mean or higher levels. Furthermore, prenatal stress was associated with lower inhibitory control only at lower levels of maternal sensitivity, but not at higher levels. Maternal sensitivity and an emotionally supportive and cognitively stimulating home environment in early childhood may be important factors that mitigate risk for mental health problems among children exposed to prenatal stress.

## Introduction

The prenatal period is characterized by a rate of neural and organ development unparalleled by any other developmental period and, consequently, heightened fetal sensitivity to environmental inputs (Barker et al., 1993; Barker, 2007; Davis et al., 2018; Gluckman et al., 2010; Howland et al., 2017). Decades of evidence within the Developmental Origins of Health and Disease model demonstrates that the in utero environment shapes life span mental and physical health (Davis et al., 2018; Monk et al., 2019; O’Donnell & Meaney, 2017). For example, maternal stress and psychological distress during pregnancy are associated with greater offspring risk for externalizing problems, internalizing problems, and psychiatric disorders extending through adulthood (Davis et al., 2018; Rogers et al., 2020; van den Bergh et al., 2017). Such evidence of enduring effects of prenatal stress on offspring risk underscores the importance of identifying postnatal environmental factors that may ameliorate effects of prenatal stress on offspring outcomes. The current study tests whether maternal sensitivity and the home environment in early childhood modify associations of prenatal stress with child behavior at age 5.

A developmental psychopathology perspective emphasizes diversity in both process and outcome and recognizes there are multiple contributors and pathways to adaptive and maladaptive outcomes (Cicchetti & Rogosch, 1996; Cicchetti & Toth, 2009; Masten & Cicchetti, 2010). Within this framework, developmental cascades refer to the cumulative consequences of many interactions and transactions within a developmental system on outcomes that alter the course of the development (Masten & Cicchetti, 2010). Developmental cascades are particularly useful to consider within the context of prenatal stress given the diversity of outcomes and developmental trajectories following prenatal stress (Hentges et al., 2019; Huizink & de Rooij, 2018; Swales et al., 2022). For example, prenatal stress has been associated with a range of forms of psychopathology (e.g., attention-deficit/hyperactivity disorder, major depression, and schizophrenia) but many children exposed to prenatal stress do not go on to develop behavior problems or psychopathology (Monk et al., 2019; Rogers et al., 2020; van den Bergh et al., 2017). Prenatal stress is theorized to influence developmental trajectories in part through alterations to offspring neural and physiological systems relevant to mental health outcomes, such as the hypothalamic–pituitary–adrenal axis, immune system, and central nervous system (Buss et al., 2011, 2012; Charil et al., 2010; Davis et al., 2018; Howland et al., 2017; Nazzari et al., 2020; Nazzari & Frigerio, 2020). However, these same systems continue to develop rapidly in the first years of life and are highly susceptible to postnatal environmental influences (Callaghan & Tottenham, 2016; Gee & Cohodes, 2021; Koss & Gunnar, 2018). Accordingly, aspects of the postnatal environment during infancy and childhood may be integral to shaping developmental trajectories following prenatal stress.

Early caregiving is one aspect of the postnatal environment that may serve a critical role in contributing to heterogeneity in outcomes following prenatal stress. Evidence from experimental animal models provides causal evidence that manipulations of postnatal maternal care offset the effects of prenatal stress on offspring stress regulation, brain development, and cognition. For example, postnatal handling counteracted effects of prenatal stress-induced deficits in hippocampal neurogenesis and behavioral abnormalities in rodents (Lemaire et al., 2006; Wakshlak & Marta, 1990). A small but growing number of studies in humans corroborate these findings from animal models. In a sample of 47 mother–infant pairs, infants of mothers diagnosed with prenatal depression had higher cortisol in the context of insensitive caregiving behaviors but not in the context of sensitive caregiving behaviors (Kaplan et al., 2008). More recently, maternal positive regard during a mother–child interaction moderated the association between prenatal stress and cognitive development in a sample of 162 preschool aged children (Schechter et al., 2017). That is, prenatal stress was associated with lower cognitive abilities in preschoolers at low levels of maternal positive regard, but not at higher levels. Similarly, prenatal stress was associated with higher negative affect and less favorable cognitive outcomes in toddlers at low levels of maternal sensitivity during a mother–infant interaction but not at high levels of maternal sensitivity (*n* = 156; Grande et al., 2021).

Collectively, this cross-species evidence demonstrates that postnatal caregiving, specifically early caregiving characterized by warmth, responsiveness, and sensitivity, mitigates the effects of prenatal stress on offspring outcomes. However, studies that test whether postnatal caregiving behaviors modify associations between prenatal stress and offspring outcomes have also largely focused on parenting behaviors during infancy (Grande et al., 2021; Kaplan et al., 2008) or self-report measures of parenting in early childhood (Clayborne et al., 2021), besides one exception that examined observed parenting behaviors during toddlerhood (Schechter et al., 2017). As a result, it is unclear whether infancy is a sensitive period when caregiving can ameliorate negative effects of prenatal stress or whether caregiving behavior during later developmental periods can also modify effects of prenatal stress on child functioning.

Furthermore, most studies to date have focused on the role of specific maternal parenting behaviors in ameliorating the consequences of prenatal stress. Nonetheless, other aspects of the caregiving environment beyond the mother–child dyad may also be protective for children, particularly the availability of emotional support and cognitive stimulation in the home environment. Bronfenbrenner’s Ecological Systems Model proposes that development is shaped by multiple levels of environmental influences and interactions between levels of influence (Bronfenbrenner & Morris, 2007). According to this model, the home environment is the most proximal influence on child outcomes given that social interactions and daily activities within the home are a primary context in which development unfolds. Measures of the home environment encompass both social and physical aspects of the home within and beyond the parent–child dyad, including the availability of emotional support (e.g., parental responsiveness; frequency of interactions with other family members) and cognitive stimulation (e.g., presence of books, toys, and music; Bradley, 2015; Frankenburg & Coons, 1986). More emotional support and cognitive enrichment in the home has been associated with more favorable cognitive abilities, executive function, and mental health in children (Eamon, 2000; Einziger et al., 2019; Lai et al., 2017; Pachter et al., 2006; Rijlaarsdam et al., 2013; Schmiedeler et al., 2014). Furthermore, an emotionally supportive and cognitively stimulating home environment has also been found to ameliorate risk for socioemotional problems among infants born preterm (Treyvaud et al., 2012). However, studies have yet to directly test whether the early childhood home environment modifies associations of prenatal stress with child outcomes. Future research in this area could help to inform additional areas of intervention beyond specific parenting behaviors.

## The current study

The current study evaluates whether maternal sensitivity and the home environment in early childhood moderate associations between prenatal stress and child behavior at age 5 in a predominantly low socioeconomic status and racially and ethnically diverse sample of 127 mother–child pairs. Specifically, we test whether these postnatal environmental factors modify links between prenatal stress and three key outcomes of (1) internalizing behaviors, (2) externalizing behaviors, and (3) inhibitory control (Oldehinkel & Ormel, 2023; Zelazo, 2020). Motivated by transdiagnostic approaches that prioritize identification of risk factors for psychopathology across diagnostic criteria (e.g., Cuthbert & Insel, 2013), we examine inhibitory control in addition to internalizing and externalizing behaviors. Within this lens, deficits in inhibitory control may serve as a transdiagnostic risk factor for psychopathology based on evidence of connections between inhibitory control and multiple forms of psychopathology (Diamond, 2013; Hodel, 2018; Zelazo, 2020). Inhibitory control is a domain of executive function that reflects the ability to inhibit dominant thought processes or actions that are not relevant to the task at hand in favor of subdominant, adaptive responses (Zelazo, 2020).

We hypothesized that higher prenatal stress would be associated with more internalizing and externalizing behaviors and lower inhibitory control. However, we hypothesized that maternal sensitivity and the home environment would modify associations between prenatal stress and child mental health and inhibitory control. Specifically, we predicted that prenatal stress would be associated with significantly more internalizing and externalizing behaviors and lower inhibitory control at lower levels of maternal sensitivity and lower levels of emotional support and cognitive stimulation in the home environment, but not at higher levels.

## Methods

### Participants and procedure

Participants in this study were 127 mother–child pairs enrolled in the multi-site Community Child Health Network (CCHN). Participants were recruited following a birth (index birth) in five study sites (Washington, DC, Baltimore, MD, Los Angeles County, CA, Lake County, IL, and North Carolina) and followed through a subsequent pregnancy, birth, and postpartum period. Participants enrolled at three eligible CCHN study sites (Washington, DC, Lake County, IL, or North Carolina) were invited to enroll in a follow-up study of the subsequent child’s development (hereafter, study child) and complete additional home visits when the study child was between 3–5 years old. The first early childhood study visit occurred when the study child was approximately four years of age (*M* = 3.85 years, *SD* = 0.52, range = 3.35–5.48) and the second visit approximately one year later (*M* = 5.07, *SD* = 0.46, range = 4.31–6.11). Trained research staff conducted structured interviews during in-home visits. The study was approved by the Institutional Review Boards at each site. Mothers provided written and informed consent for themselves and their children. Participants were compensated for study visits.

The current sample includes all participants enrolled in the follow-up study (*n* = 127), and data from study visits during the subsequent pregnancy and early childhood of the study child. Mean maternal age was 34 years (*M* = 33.98, *SD* = 5.48) at the first early childhood visit. Participants identified as Hispanic/Latina (48.8%), non-Hispanic White (31.5%), and Black/African American (21.3%). Mean per capita income adjusted for cost of living at each study site was $12,604 (*SD* = $12,725, range = $22.08–$70,000), and the modal level of educational attainment was high school diploma or GED (40.2%; M years of education = 12.72, *SD* = 3.57, range = 6–21). Approximately half of the mothers were married to the study child’s father (46.4%) at the early childhood study visits and the rest were cohabiting but not married (30.7%) or single (23.6%). Just over half of the study children were girls (53.5%) and about half were the second-born child (51.2%). All study children were singleton births. The majority of participants were enrolled at the study site in Chicago, IL (77.1%).

### Measures

#### Perceived stress

Prenatal stress was operationalized as maternal perceived stress in pregnancy to capture maternal stress appraisals across pregnancy. Perceived stress was measured with the 10-item version of the Perceived Stress Scale in which participants rate the stressfulness of situations in the past month (Cohen et al., 1983). Participants report on the degree to which they find their lives to be unpredictable, uncontrollable, and overwhelming on a 5-point Likert scale from 0 (*never*) to 4 (*almost always*). The Perceived Stress Scale is an established index of general stress appraisal that is commonly used and validated for use during pregnancy (Karam et al., 2012). Mothers reported on perceived stress twice in pregnancy during the second and third trimester; scores at each pregnancy visit were positively correlated and therefore were scored as an average for primary analyses (*r* = 0.56, *p* < .001). Mothers also reported on perceived stress at both early childhood visits.

#### Maternal sensitivity

Maternal sensitivity was scored from observed maternal parenting behaviors during a semi-structured play task at the first early childhood visit. During the task, mothers and children were offered toy boxes and instructed to play with the toys for 15 minutes in mothers’ language of preference (69% English, 31% Spanish). Trained coders scored parenting behaviors along seven dimensions (sensitivity to non-distress, positive regard, stimulation of cognitive development, intrusiveness, negative regard, detachment, and flatness of affect) using the validated 36-month mother–child interaction coding system from the National Institute of Child Development (NICHD) Study of Early Childcare and Youth Development (1993). Each parenting dimension was scored globally on a scale of 1 (*not at all characteristic*) to 4 *(highly characteristic*). Two coders were monolingual English-speakers and one coder was a bilingual English and Spanish speaker. Videos in Spanish were coded by the bilingual coder (*n* = 32) or translated for coding when that was not possible (*n* = 4). Mean ratings of maternal sensitivity did not significantly differ for mothers coded in English (*M* = 9.08, *SD* = 1.41) versus Spanish (*M* = 8.87, *SD* = 1.78; *p* = .55). All coders were blind to other data gathered on study participants. To assess reliability, 24 percent of the videos were randomly selected and coded independently by an expert coder to obtain an index of inter-rater reliability (mean percent agreement across subscales = 94%; range = 81%–100%).

To be consistent with prior research using the same coding system and evaluating the role of maternal sensitivity in moderating the links between prenatal stress and offspring outcomes (Grande et al., 2021), maternal sensitivity was scored as a composite of maternal sensitivity to non-distress (*being attuned to and centered on the child and letting the child guide the play*), maternal positive regard (*warmth, praise, physical affection*), and reverse-coded maternal intrusiveness (*adult-centered, controlling the interaction, imposing the parent*’*s agenda regardless of child signals*) (α = 0.76).

#### Early childhood home environment

Mothers reported on the home environment at the second early childhood visit with an adapted version of the Home Screening Questionnaire (HSQ) 3–6-year form.^1^ The HSQ was developed from the Home Observation for Measurement of the Environment to provide an objective measure of the home environment and improve the early identification of children who will have school problems. Whereas the Home Observation Measurement of the Environment takes over an hour to complete, the HSQ takes 15–20 minutes to complete (Frankenburg & Coons, 1986). Mothers responded to 34 items that measure the home environment at in-home interviews, including both multiple choice, fill-in-the blank, and yes/no questions. Specifically, the HSQ asks participants to report on items pertaining to the availability of emotional support, such as how household members approach discipline, foster independence, and spend time with the child (e.g., “What would you do if your child got angry and hit you?”; “How often do you and your child get a chance to play together?”), and cognitive stimulation, including the degree to which the home environment provides opportunities for literary, scientific, and mathematical development (e.g., “Approximately how many children’s books does your family own?”; “How often does someone get a chance to read stories to your child?”). The HSQ yields one total score that rates the overall home environment (total possible scores = 0–42), with higher scores indicating a home environment characterized by greater emotional support and cognitive stimulation. The HSQ is validated for use across socioeconomically and culturally diverse samples (Bradley, 2015).

#### Child externalizing and internalizing behaviors

Child externalizing and internalizing behaviors were measured with the Child Behavior Checklist (CBCL)1.5–5 year version at the second early childhood visit. The CBCL is a validated 100-item parent-report checklist that assesses emotional and behavioral problems in children. Parents are asked to rate how true each item is for their child in the past two months on a scale of 0 (*not true*) to 2 (*very true*). The measure can yield scores for two major subscales: internalizing and externalizing. Higher scores reflect greater presence and severity of symptoms. Consistent with standard practice, scores were transformed into T-scores, with scores between 65 and 69 indicating risk for clinically significant behavioral problems and scores greater than 70 indicating clinically significant behavioral problems (Achenbach & Rescorla, 2000).

#### Child inhibitory control

Child inhibitory control, or the ability to inhibit or block automatic or inappropriate responses (Posner & Rothbart, 2000; Zelazo, 2020), was measured using the Early Childhood version of the NIH Toolbox Cognition Battery (ages 3–6; Weintraub et al., 2013) at the second early childhood visit. Inhibitory control was assessed with the Flanker Inhibitory Control and Attention Task. In the Flanker, children indicate the orientation of a centrally presented stimulus while inhibiting their attention to the surrounding stimuli (flankers) across three test blocks (total administration time = 3–4 minutes). The NIH Toolbox has excellent reliability and validity in Spanish and English (Weintraub et al., 2013). About three-quarters (77.4%) of children in the current sample completed the assessments in English, and there were no significant differences in scores by assessment language. Domains are scored using fully corrected T-scores adjusting for child age, sex, race/ethnicity, and maternal education.

#### Sociodemographic and medical variables

Participants reported their age, education, household income, household size, racial/ethnic identity, relationship status, and number of previous live births at the time of enrollment in CCHN. Updates to income and relationship status were collected at each study visit. Per capita income was calculated as household income divided by household size, adjusted for cost of living in each study site. Child birth weight and gestational age at birth were extracted from medical records.

#### Data analytic plan

Analyses were conducted in SPSS (IBM Corp.) and R (R Core Team, 2021). Primary study variables were examined for normality and outliers (> 4 standard deviations from sample mean) prior to analysis. All primary study variables were within typical range for normality (skewness < 2 and kurtosis < 7) and within 4 standard deviations of the sample mean. A series of multiple linear regression models were used to test primary research questions that included an interaction term between prenatal stress and (1) maternal sensitivity and (2) the home environment. Each child outcome (externalizing, internalizing, inhibitory control) and moderator was evaluated in separate models; thus, there were six total models. Simple slopes were used to probe interactions at one standard deviation below the sample mean, at the sample mean, and one standard deviation above the sample mean for interaction terms *p* < .05. If interaction terms were not significant, we interpreted main effects.

In accordance with modern missing data recommendations, missing data were handled using full information maximum likelihood. Full information maximum likelihood is recommended when missing data exceeds 10% to reduce bias of estimates, increase power, and strengthen generalizability of results and is also appropriate in the context of quantitative moderators (Dong & Peng, 2013; Enders, 2010; Madley-Dowd et al., 2019). Missing data on primary study variables ranged from 7.8% (maternal sensitivity) to 25.2% (child inhibitory control). Rates of missingness across all study variables are presented in Supplemental Table 1. Missing data was primarily attributable to missed study visits during the longitudinal study spanning many years or missing data on parts of interviews within each visit (e.g., participant declined to complete certain measures).

#### Covariates

Primary analyses adjusted for covariates. We included maternal perceived stress at the time child behavior was measured as a covariate to isolate the unique effects of prenatal stress on child mental health and inhibitory control. In the current sample, concurrent maternal perceived stress was inversely associated with home environment quality (*r* = −0.28, *p* = .01) and maternal sensitivity (*r* = −0.19, *p* = .04) and positively associated with prenatal stress (*r* = 0.34, *p* < .001), child internalizing (*r* = 0.27, *p* = .01), and child externalizing (*r* = 0.29, *p* = .003).

Per capita income, mother education (years), language preference, relationship status, birth order (i.e., number of children), child age, child biological sex, gestational age, and birth weight were evaluated as additional covariates and included in final models if significantly associated with primary study variables (*p* < .05). Number of children was positively related to internalizing (*r* = 0.20, *p* = .04) and externalizing behaviors (*r* = 0.21, *p* = .03). Per capita income was inversely associated with prenatal stress (*r* = −0.27, *p* = .01) and internalizing behaviors (*r* = −0.30, *p* = .003) and positively associated with home environment quality (*r* = 0.45, *p* < .001). Married participants reported significantly higher prenatal stress than participants who were in a relationship or single (F[2, 99] = 9.65, *p* < .001), and significantly higher home environment quality than participants who were in a relationship or single (F[2,105] = 4.70, *p* = .01). Thus, number of children (dummy-coded; two children vs. two or more children), per capita income, and concurrent maternal perceived stress and relationship status (dummy-coded as married vs. single or in a relationship but not married) were included as covariates in final models.

## Results

### Descriptive statistics

Table 1 presents descriptive statistics and bivariate associations of primary study variables. Mean levels of emotional support and cognitive stimulation in the home environment were high (*M* = 29.63, *SD* = 5.72), but there was considerable variability (range = 10–38). Similarly, maternal sensitivity was high on average (*M* = 9.05, *SD* = 1.70) and varied within the sample (range = 5–12). There was also a wide range of scores for internalizing and externalizing behaviors (internalizing *M* = 52.45, *SD* = 11.60, range = 29–77; externalizing *M* = 49.46, *SD* = 11.33, range = 28–71). Ten percent of children were at risk for clinically significant internalizing behaviors and 7% of children were at risk for clinically significant externalizing behaviors. A small proportion of the sample was rated as having clinically significant internalizing and externalizing (1% and 2%, respectively), consistent with rates in other community samples (e.g., Cents et al., 2013; Sharp et al., 2015). As shown in bivariate associations (see Table 1), higher prenatal stress was associated with more child internalizing behaviors. Additionally, maternal sensitivity and the home environment were moderately positively associated and were associated with lower child internalizing and externalizing behaviors.

### Primary analyses

In primary analyses, we tested interaction effects of prenatal stress, maternal sensitivity, and the home environment on child externalizing, internalizing, and inhibitory control. Simple slopes values for simple slopes analyses corresponded to values of 7.35 (−1 SD below mean), 9.05 (sample mean), and 10.75 (+1 SD above mean) for maternal sensitivity and values of 23.91 (−1 SD below mean), 29.63 (sample mean), and 35.35 (+1 SD above mean) for the home environment. Complete regression coefficients with standard errors and confidence intervals are presented in Tables 2 and 3.

### Prenatal stress and child outcomes: moderation by maternal sensitivity

We first tested whether maternal sensitivity during the parent–child interaction modified the association between prenatal stress and child externalizing behaviors, internalizing behaviors, and inhibitory control.

#### Child externalizing

There was a significant main effect of maternal sensitivity on child externalizing such that higher maternal sensitivity was associated with fewer child externalizing behaviors (*β* = −0.235, *B* = −1.517, *SE* = 0.697, *p* = .030). There was no significant main effect of prenatal stress on child externalizing, nor was there a significant interaction effect of prenatal stress and maternal sensitivity on child externalizing (all *p*’s > .657).

#### Child internalizing

**Figure 1.**
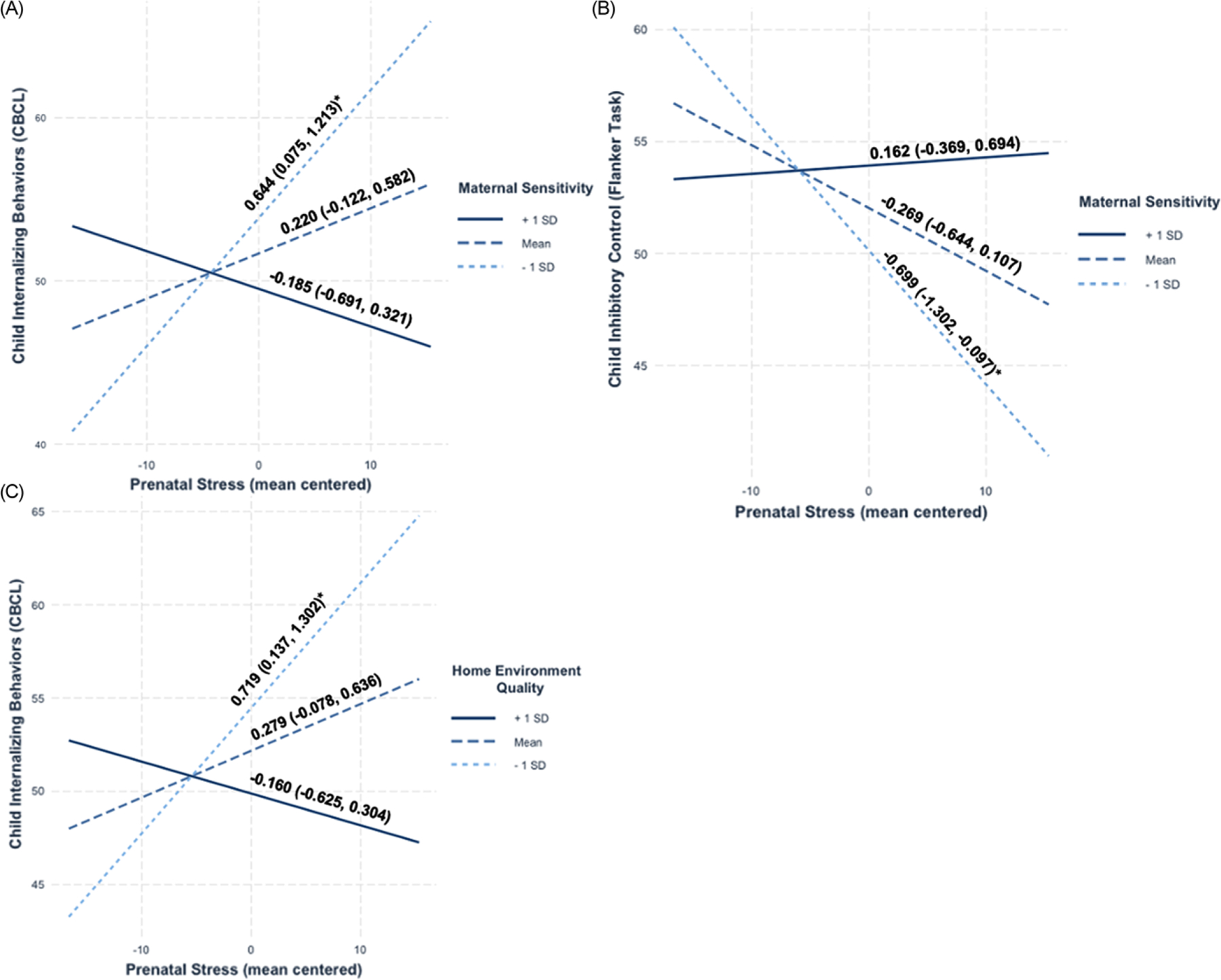
Simple slopes analysis.

There was a significant main effect of maternal sensitivity on child internalizing such that higher maternal sensitivity was associated with fewer child internalizing behaviors (*β* = −0.249, *B* = −1.662, *SE* = 0.701, *p* = .018). Prenatal stress was not associated with child internalizing behaviors, but there was an interaction between prenatal stress and maternal sensitivity on child internalizing (*β* = −0.226, *B* = −0.272, *SE* = 0.135, *p* = .044). Simple slopes analyses revealed that prenatal stress was associated with significantly more child internalizing behaviors at low levels of maternal sensitivity in the current sample (*β* = 0.361, *B* = 0.644, *SE* = 0.290, *p* = .027) but was not significantly associated with child internalizing at average (*β* = 0.135, *B* = 0.220, *SE* = 0.180, *p* = .201) or high levels of maternal sensitivity (*β* = −0.091, *B* = −0.185, *SE* = 0.258, *p* = .474). Simple slopes are shown in Figure 1a.

#### Child inhibitory control

The main effects of prenatal stress or maternal sensitivity on child inhibitory control were not statistically significant (all *p*’s > .169); however, there was a significant interaction effect of prenatal stress and maternal sensitivity on child inhibitory control (*β* = 0.250, *B* = 0.283, *SE* = 0.142, *p* = .046). Specifically, prenatal stress was associated with significantly lower child inhibitory control at low levels of maternal sensitivity (*β* = −0.416, *B* = −0.699, *SE* = 0.307, *p* = .023), but not at average (*β* = −0.166, *B* = −0.269, *SE* = 0.192, *p* = .161) or high levels of maternal sensitivity (*β* = 0.083, *B* = 0.162, *SE* = 0.271, *p* = .550; see Fig. 1b).

### Prenatal stress and child outcomes: moderation by home environment

Next, we tested whether the early childhood home environment modified the association between prenatal stress and child externalizing, internalizing, and inhibitory control.

#### Child externalizing

There was a significant main effect of the home environment on child externalizing such that a home environment characterized by higher levels of emotional support and cognitive stimulation was associated with less child externalizing behaviors (*β* = −0.320, *B* = −0.598, SE = 0.209, *p* = .004). The main effect of prenatal stress on child externalizing was not significant, nor was there an interaction between prenatal stress and the home environment on child externalizing (all *p*’s > .661).

#### Child internalizing

There was also a significant main effect of the home environment on child internalizing such that a home environment characterized by higher levels of emotional support and cognitive stimulation was associated with fewer child internalizing behaviors (*β* = −0.290, *B* = −0.546, *SE* = 0.205, *p* = .009). However, there was also a significant interaction between prenatal stress and home environment on child internalizing (*β* = −0.220, *B* = −0.083, *SE* = 0.037, *p* = .024). Prenatal stress was associated with more child internalizing behaviors at low levels of emotional support and lower cognitive stimulation in the home environment (*β* = 0.388, *B* = 0.719, *SE* = 0.297, *p* = .015), but not at average (*β* = 0.167, *B* = 0.279, *SE* = 0.182, *p* = .125) or high levels (*β* = −0.053, *B* = −0.160, *SE* = 0.237, *p* = .498; see Fig. 1c).

#### Child inhibitory control

The main effects of prenatal stress and home environment on child inhibitory control was not significant, nor was there a significant interaction effect of prenatal stress and the home environment on inhibitory control (all *p*’s > .158).

## Discussion

A developmental psychopathology approach recognizes that there are multiple pathways to adaptive and maladaptive outcomes (e.g., Cicchetti & Rogosch, 1996; Cicchetti & Toth, 2009; Masten & Cicchetti, 2010; Masten, 2006). Identifying the postnatal environmental factors that shape developmental cascades following prenatal stress is critical given evidence that prenatal stress can increase risk for psychopathology through adulthood. However, research in this area is limited in humans. In the current study, we aimed to address this research gap by testing whether two measures of the caregiving environment in early childhood, maternal sensitive parenting behaviors and availability of cognitive stimulation and emotional support in the home environment, modified associations between prenatal stress with child mental health and inhibitory control.

Consistent with study hypotheses, maternal sensitivity and the early childhood home environment moderated associations between prenatal stress and child internalizing behaviors and inhibitory control. Specifically, prenatal stress was associated with more child internalizing behaviors at low levels of maternal sensitivity and low levels of emotional support and cognitive stimulation in the home environment but was not associated with internalizing behaviors at average or high levels. Furthermore, prenatal stress was associated with lower inhibitory control at low levels of maternal sensitivity but not at average or high levels of maternal sensitivity. Prenatal stress was not associated with externalizing behaviors, nor did prenatal stress or early childhood caregiving environment interact to predict externalizing behaviors; however, higher maternal sensitivity and a home environment characterized by higher emotional support and cognitive stimulation were associated with fewer externalizing behaviors. This pattern of results was robust in controlled analyses with relevant covariates, specifically sociodemographic characteristics and concurrent maternal perceived stress. These results add to a growing body of evidence in humans that postnatal caregiving ameliorates the effects of prenatal stress on offspring outcomes and advances understanding of which postnatal environmental factors help protect against effects of prenatal stress. Importantly, these findings can inform postnatal opportunities for prevention and intervention to counteract risks associated with prenatal stress.

The Developmental Origins of Health and Disease model proposes that the in utero environment sets probabilistic parameters for health and well-being across the life span (Barker, 2007; Bateson et al., 2014; Gluckman et al., 2005). Consistent with this model, many empirical studies have reported that prenatal stress (conceptualized and measured in various ways) is associated with greater risk for internalizing symptoms in offspring (Betts et al., 2014; Davis et al., 2018; Monk et al., 2019; van den Bergh et al., 2017). Consistent with these prior findings, prenatal stress was positively associated with internalizing behaviors in early childhood in the current study. Importantly, however, high maternal sensitivity and a home environment with high emotional support and cognitive stimulation ameliorated effects of prenatal stress on offspring internalizing behaviors in this study. These results are consistent with causal evidence from animal models and add to growing evidence in humans that the effects of prenatal stress are modifiable by postnatal caregiving influences (Grande et al., 2021; Kaplan et al., 2008; Lemaire et al., 2006; Schechter et al., 2017; Wakshlak & Marta, 1990).

The current study further advances understanding of postnatal influences that modify effects of prenatal stress by examining aspects of the caregiving environment beyond parenting behaviors and doing so in early childhood, as most previous studies have focused on specific parenting behaviors, often during infancy. Of note, consistent with Bronfenbrenner’s Ecological Systems Model, the present findings suggest that other aspects of the caregiving environment in addition to specific maternal behaviors attenuate risk after prenatal stress (Bronfenbrenner & Morris, 2007). Collectively, these results offer several potential avenues for intervention, both within the parent–child dyad and in the broader home environment. Specifically, implementation of existing evidence-based parenting interventions (e.g., ATTACH Intervention; Parent-Child Interaction Therapy) and home visiting programs (e.g., Early Health Start; Nurse-Family Partnership) may help to mitigate offspring risk via changes to parenting behaviors and the home environment (Letourneau et al., 2020; Somers et al., 2024; Sullivan et al., 2023). Prior work demonstrates that parenting interventions and home visiting improves maternal sensitivity and the quality of the home environment (e.g., Kendrick et al., 2000; Sierau et al., 2016; Thomas & Zimmer-Gembeck, 2011). To further inform prevention and intervention efforts, future research could extend the present study by testing whether parenting behaviors and the home environment may mitigate offspring risk in clinical samples. Only a small proportion of the children in the present community sample showed clinically significant internalizing or externalizing behaviors.

There are several ways maternal sensitivity and an emotionally supportive and cognitively stimulating home environment could protect against offspring risk for internalizing after prenatal stress. First, prenatal stress is thought to influence offspring risk for internalizing through several pathways, including alterations to neurodevelopment (Buss et al., 2011, 2012; Charil et al., 2010; Davis et al., 2018; Lautarescu et al., 2020; Sandman et al., 2011), hypothalamic–pituitary–adrenal axis regulation (Howland et al., 2017; Rinne et al., 2022), and immune system functioning (Hantsoo et al., 2019; Nazzari et al., 2020; Nazzari & Frigerio, 2020). These same systems continue to develop rapidly in the first years of life and are highly sensitive to environmental inputs through early childhood (Callaghan & Tottenham, 2016; Gee & Cohodes, 2021; Gunnar & Donzella, 2002; Gunnar & Quevedo, 2007; Koss & Gunnar, 2018). Thus, sensitive parenting behaviors and an emotionally supportive and cognitively stimulating home environment in early childhood may alter neurodevelopment and physiological functioning in a manner that mitigates risk for internalizing following prenatal stress. Second, a supportive and responsive caregiving environment in the first years of life may help to foster secure attachment bonds, emotional security, and emotion regulation even in contexts of risk (Chimed-Ochir et al., 2022; Crespo et al., 2017; Glynn et al., 2021). For example, according to Emotional Security Theory, a family environment that is warm and responsive may promote feelings of emotional safety and emotion regulation skills that in turn prevent internalizing symptoms (Cummings & Miller-Graff, 2015; Maccoby, 2000). Future studies could evaluate the mechanistic pathways through which postnatal caregiving may mitigate risk, such as epigenetic modifications and attachment-related processes (Conradt et al., 2018; Murgatroyd et al., 2015).

In contrast to the finding that both maternal sensitivity and the home environment modified associations between prenatal stress and child internalizing, only maternal sensitivity, not the home environment, modified associations between prenatal stress and child inhibitory control. Prenatal stress has been previously associated with deficits in inhibitory control, a domain of executive function that is associated with transdiagnostic psychopathology (Davis et al., 2018; Posner & Rothbart, 2000; Schlotz et al., 2008; Zelazo, 2020). Rapid development of inhibitory control occurs in the preschool years, primarily within the context of the parent–child relationship (Carlson & Wang, 2007; Fay-Stammbach et al., 2014; Feldman, 2007). Accordingly, specific caregiver behaviors may be especially salient to the development of these skills. For example, in the first years of life, children largely rely on caregivers to help regulate behavior, emotions, and physiology through external regulation and co-regulation as their self-regulatory skills are still developing (Feldman, 2007; Gartstein et al., 2013; Posner & Rothbart, 2000). Indeed, sensitive caregiving has been associated with inhibitory control and greater increases in inhibitory control over the preschool years independently of contextual factors and socioeconomic status (Carlson & Wang, 2007; Geeraerts et al., 2021; Ku & Blair, 2023; Martinez-Torteya et al., 2014; Morris et al., 2017). Thus, specific maternal caregiving behaviors characterized by warmth, positivity, and responsiveness can both help children to regulate emotions, behavior, and cognition as well as provide a model of appropriate self-regulatory strategies, regardless of broader environmental factors like the home environment.

Contrary to hypotheses, prenatal stress was not associated with externalizing behaviors, nor did prenatal stress interact with postnatal environmental factors to predict externalizing behaviors. These results differ slightly from recent work showing that self-reported parent involvement can attenuate the effects of prenatal stress on externalizing symptoms in 8-year-old girls, but not boys, in a large cohort study of mother–child dyads (Clayborne et al., 2021). Different measures of parenting behaviors (self-report vs. observed) and age of behavior problem measurement in children (age 8 vs. age 5) may contribute to these differences across studies. Additionally, the current study was underpowered to evaluate sex differences. Nonetheless, consistent with past research, positive parenting behaviors and home environments in early childhood were associated with fewer externalizing behaviors in children in the current study (Anderson & Goodnight, 2022; Boeldt et al., 2012; Chimed-Ochir et al., 2022; Hentges et al., 2018; Pinquart, 2017; Shaw et al., 1998; Trentacosta & Shaw, 2008). These results align with theoretical models that state that early caregiving environment is critical to the expression of these externalizing types of behavior problems in children (Cummings & Miller-Graff, 2015; Mikulincer & Shaver, 2019; Patterson, 2016). Within this context, a caregiving environment with higher sensitivity, support, and cognitive stimulation may promote emotional security and internalization of regulatory processes in children that protects them from developing externalizing behavior problems independently of degree of prenatal stress (Bandura, 1976; Cummings & Miller-Graff, 2015). In the current study, for example, the bivariate association between prenatal stress and externalizing behaviors was similar to the effect size reported in a recent meta-analysis on prenatal stress and child externalizing (Tung et al., 2023). However, the main effect of prenatal stress on externalizing was substantially smaller when postnatal environmental factors were included in the model, suggesting that maternal sensitivity and the home environment may be particularly relevant to the development of externalizing behaviors. Based on the present results, comprehensive assessment of prenatal stress and postnatal environmental factors in future research may help further understanding of the factors that most strongly contribute to the emergence of externalizing behaviors.

Future research could aim to explain why there were interactive effects of prenatal stress and postnatal caregiving on some child outcomes (internalizing, inhibitory control), but not others (externalizing). Consistent with the current pattern of findings, another recent large cohort study found that positive maternal mental health moderated the effect of prenatal stress on child internalizing behaviors, but not externalizing behaviors (Clayborne et al., 2023). Examining the biological (neuroendocrine and immune) and behavioral mechanisms through which postnatal caregiving mitigates offspring risk for mental health problems following prenatal stress may be an avenue for explaining this pattern of results (Callaghan & Tottenham, 2016; Conradt et al., 2018; Davis et al., 2018; Hantsoo et al., 2019). Of note, deficits in inhibitory control are a transdiagnostic risk factor for subsequent psychopathology (Zelazo, 2020). Therefore, it is possible that inhibitory control could be a mechanism connecting the interactive effect of prenatal stress and maternal sensitivity on subsequent child mental health problems. For example, inhibitory control was inversely associated with both internalizing and externalizing behaviors in children in the present sample. The current study was unable to directly test this mechanistic pathway due to sample size and study design (i.e., concurrent assessment of child outcomes); however, this could be a productive pathway to examine in future longitudinal studies designed with repeated measures of child inhibitory control, internalizing behaviors, and externalizing behaviors.

### Strengths and limitations

The current study has notable strengths including the longitudinal design with study visits occurring in pregnancy through early childhood. Additionally, all assessments took place in homes, a more naturalistic setting, and were administered by trained researchers and using well-validated measures and standardized procedures. The sample was diverse with respect to race and ethnicity and included participants who are historically under-represented in child development research. Developmental outcomes were assessed by both maternal report and standardized assessments reducing shared method bias. The use of reliably coded measures of maternal parenting behaviors is another strength as it reduces potential bias associated with self-report parenting measures.

Some limitations also warrant mention. Mean levels of behavioral problems were low on average, although they varied in the present sample; nonetheless, it is not clear whether these results would generalize to clinical samples. Also, it was outside of the scope of the current study to look at sex-specific associations. This may be an important area of future research given evidence of sex-specific fetal programming effects (Clayborne et al., 2021). Additionally, mothers reported on the home environment at the same time child outcomes were assessed, which may result in shared method variance with maternal reports of child mental health using the CBCL and precludes any conclusions about prospective, temporally sensitive associations between the home environment and subsequent child outcomes. Finally, although the HSQ is a shorter measure than the original Home Observation Measurement of the Environment scale that is more feasible to include in community studies and long protocols, the HSQ does not yield separable subscales for emotional support and cognitive stimulation like the original measure. Future research could test whether there are unique moderation effects for different aspects of the home environment (emotional support, cognitive stimulation) using the full Home Observation Measurement of the Environment scale or other measures of the home environment that separately measure these dimensions (Grieve & Richter, 1990).

## Conclusion

Evidence in support of the Developmental Origins of Health and Disease model demonstrates enduring effects of prenatal stress on offspring outcomes across the life span. Importantly, the current findings add to small but growing evidence in humans that postnatal environmental factors may mitigate risks associated with prenatal stress. Specifically, sensitive parenting behaviors and an emotionally supportive and cognitively stimulating home environment may reduce risk for internalizing behaviors and lower inhibitory control following prenatal stress. Accordingly, interventions that promote positive parenting behaviors and an engaging and supportive home environment may reduce the risk of mental health problems among children exposed to prenatal stress.

## Supplementary Material

Supplementary Materials

## Figures and Tables

**Table 1.** Descriptive statistics and bivariate associations of primary study variables

Variable	*M* (*SD*)	Range	1	2	3	4	5	6
1. Prenatal stress	16.66 (6.73)	0 − 32	––					
2. Maternal sensitivity	9.05 (1.70)	5 − 12	− 0.27*	––				
3. Home environment	29.63 (5.72)	10 − 38	− 0.27*	0.48***	––			
4. Child internalizing	52.45 (11.60)	29 − 77	0.24*	− 0.31**	− 0.35***	––		
5. Child externalizing	49.46 (11.33)	28 − 71	0.15	− 0.23*	− 0.28**	0.63***	––	
6. Child inhibitory control	51.55 (10.53)	14 − 74	− 0.13	0.11	− 0.12	− 0.19^	− 0.26*	––

*** *p* < .001

** *p* < .01

* *p* < .05

^ *p* < .10.

**Table 2.** Moderation by maternal sensitivity

Outcome	Predictor	*B* [95% CI]	*SE*	*β*
Child externalizing behaviors	Prenatal stress	0.079 [ − 0.270, 0.429]	0.178	0.048
	Maternal sensitivity	− 1.517 [ − 2.884, −0.150]*	0.697	− 0.235
	Prenatal stress × maternal sensitivity	− 0.033 [ − 0.302, 0.237]	0.138	− 0.028
	Income	0.076 [ − 0.100, 0.251]	0.090	0.099
	Relationship status	0.655 [ − 3.974, 5.285]	2.362	0.033
	Number of children	− 1.348 [ − 5.238, 2.542]	1.985	− 0.069
	Concurrent perceived stress	0.432 [0.076, 0.787]*	0.181	0.258
			*R* ^2^	0.135
Child internalizing behaviors	Prenatal stress	0.217 [ − 0.126, 0.559]	0.175	0.128
	Maternal sensitivity	− 1.663 [ − 3.036, −0.289]*	0.701	− 0.249
	Prenatal stress × maternal sensitivity	− 0.272 [ − 0.538, −0.007]*	0.135	− 0.226
	− *1 SD*	0.644 [0.075, 1.213]*	0.290	0.361
	*Mean*	0.220 [ − 0.122, 0.582]	0.180	0.135
	+ *1 SD*	− 0.185 [ − 0.691, 0.321]	0.258	− 0.091
	Income	− 0.170 [ − 0.345, 0.006]^	0.089	− 0.213
	Relationship status	2.296 [ − 2.154, 6.947]	2.322	0.118
	Number of children	− 1.099 [ − 4.963, 2.766]	1.972	− 0.052
	Concurrent perceived stress	0.271 [ − 0.082, 0.624]	0.180	0.157
			R^2^	0.216
Child inhibitory control	Prenatal stress	− 0.257 [ − 0.623, 0.109]	0.187	− 0.160
	Maternal sensitivity	0.691 [ − 0.687, 2.068]	0.703	0.109
	Prenatal stress × maternal sensitivity	0.283 [0.006, 0.561]*	0.142	0.250
	− *1 SD*	− 0.699 [ − 1.302, −0.097]*	0.307	− 0.416
	*Mean*	− 0.269 [ − 0.644, 0.107]	0.192	− 0.166
	+ *1 SD*	0.162 [ − 0.369, 0.694]	0.271	0.083
	Income	− 0.067 [ − 0.241, 0.106]	0.089	− 0.089
	Relationship status	0.928 [ − 3.833, 5.689]	2.429	− 0.004
	Number of children	3.257 [ − 0.755, 7.269]	2.047	− 0.169
	Concurrent perceived stress	0.124 [ − 0.237, 0.485]	0.184	0.076
			R^2^	0.133

* *p* < .05

^ *p* < .10.

**Table 3.** Moderation by home environment

Outcome	Predictor	*B* [95% CI]	*SE*	*β*
Child externalizing behaviors	Prenatal stress	0.078 [ − 0.272, 0.428]	0.179	0.047
	Home environment	− 0.598 [ − 1.007, −0.189]**	0.209	− 0.320
	Prenatal stress × home environment	− 0.008 [ − 0.081, 0.065]	0.037	− 0.021
	Income	0.124 [ − 0.049, 0.298]	0.088	0.160
	Relationship status	0.622 [ − 3.843, 5.086]	2.278	0.031
	Number of children	− 1.515 [ − 5.339, 2.308]	1.951	− 0.076
	Concurrent perceived stress	0.405 [0.055, 0.756]^	0.179	0.240
			*R* ^2^	0.178
Child internalizing behaviors	Prenatal stress	0.279 [ − 0.067, 0.626]	0.177	0.167
	Home environment	− 0.551 [ − 0.954, −0.138]**	0.205	− 0.292
	Prenatal stress × home environment	− 0.083 [ − 0.155 − 0.011]*	0.037	− 0.220
	− *1 SD*	0.719 [0.137, 1.301]*	0.297	0.388
	*Mean*	0.279 [ − 0.078, 0.636]	0.182	0.167
	+ *1 SD*	− 0.160 [ − 0.625, 0.304]	0.237	− 0.053
	Income	− 0.098 [ − 0.271, 0.074]	0.088	− 0.125
	Relationship status	1.538 [ − 2.866, 5.942]	2.247	0.077
	Number of children	− 1.280 [ − 5.113, 2.552]	1.955	− 0.064
	Concurrent perceived stress	0.264 [ − 0.086, 0.614]	0.179	0.154
			R^2^	0.198
Child inhibitory control	Prenatal stress	− 0.278 [ − 0.666, 0.109]	0.197	− 0.174
	Home environment	− 0.288 [ − 0.742, 0.167]	0.232	− 0.159
	Prenatal stress × home environment	0.021 [ − 0.067, 0.109]	0.045	0.059
	Income	− 0.069 [ − 0.243, 0.105]	0.089	− 0.092
	Relationship status	2.980 [ − 1.693, 7.653]	2.384	0.155
	Number of children	3.349 [0.727, 7.426]	1.610	0.174
	Concurrent perceived stress	0.139 [ − 0.229, 0.507]	0.232	0.059
			R^2^	0.091

** *p* < .01

* *p* < .05

^ *p* < .10.

## Footnotes

1 The present study made a few changes to the original measure to reflect current parenting practices and technology, including rewording questions to align with current understanding of child development and to include experiences related to recent advancements with technology.
