# Supplementary material for: Prospective associations of prenatal stress with child behavior: Moderation by the early childhood caregiving environment
Source: Dev Psychopathol. Author manuscript; Available in PMC 2025 Nov 1. (PMC11557737; doi:10.1017/S0954579424000920)
Supplement: Supplementary Materials [file NIHMS2012146-supplement-Supplementary_Materials.docx]

**Supplemental Materials**

**Table 1.** Rates of missingness across primary study variables

| **Variable** | **Missingness** |
| --- | --- |
| Prenatal Stress | 14.2% |
| Maternal Sensitivity | 7.8% |
| Home Environment | 19.7% |
| Child Internalizing Behaviors | 19.7% |
| Child Externalizing Behaviors | 20.5% |
| Child Inhibitory Control | 25.2% |
